# Association of prognostic nutritional index with peripheral artery disease in US adults: a cross-sectional study

**DOI:** 10.1186/s12872-024-03815-0

**Published:** 2024-03-02

**Authors:** Dikang Pan, Julong Guo, Zhixiang Su, Wenzhuo Meng, Jingyu Wang, Jianming Guo, Yongquan Gu

**Affiliations:** 1 Department of Vascular Surgery, Xuanwu Hospital, Capital Medical University, Beijing, China; 2 Renal Division, Peking University First Hospital, Beijing, China

**Keywords:** Peripheral artery disease, Prognostic nutritional index, National Health and Nutrition Examination Survey, Cross-sectional study, Risk

## Abstract

**Background:**

The objective of this study was to investigate the relationship between the prognostic nutritional index (PNI) and peripheral artery disease (PAD).

**Methods:**

The present study is a cross-sectional study based on the National Health and Nutrition Survey (1999–2004). The laboratory-calculated PNI was divided into four groups based on quartiles(Q1:PNI ≤ 50.00; Q2: 50.01–53.00; Q3:53.01–56.00; Q4: > 56.00). PAD was defined as an ankle brachial pressure index (ABPI) ≤ 0.9 on the left or right. The relationship between PNI and PAD was examined using multifactor weighted logistic regression analysis, as well as subgroup analysis. Subgroup analyses were conducted based on demographic and clinical variables.

**Results:**

A total of 5,447 individuals were included in our final analysis. The age of the participants was 59.56 ± 13.10 years, and males accounted for 52.8% (*n* = 2820). The prevalence of PAD was 6.7% (*n* = 363). After adjusting for all factors, participants with Q1 still had an increased risk of PAD, with an OR value of 1.593 and a 95% CI of 1.232–1.991. Subgroup analysis showed no significant interaction among multiple factors.

**Conclusions:**

In summary, we report that lower PNI are associated with a higher risk of PAD in US adults. It is hoped that this discovery can provide a reference for the prevention of PAD.

## Introduction

Peripheral artery disease (PAD) is a progressive disease characterized by the presence of atherosclerotic blockages in the peripheral vascular system, commonly affecting the arteries of the lower extremities. It often serves as a warning sign for underlying atherosclerotic disease elsewhere in the body’s blood vessels. People diagnosed with PAD are at a heightened risk of myocardial infarction, ischemic stroke, and cardiovascular death [1]. PAD affects 3–7% of the general population and up to 20% of individuals over the age of 75 [2]. The economic burden and health risks associated with PAD cannot be overstated. Therefore, it is crucial to identify the root causes of PAD and take appropriate actions.Malnutrition is typically defined as having a low body mass index (BMI) and low serum albumin levels [3]. It is associated with a range of metabolic disorders including steatosis, increased lipolysis and fatty acid oxidation, decreased circulating amino acids, reduced peroxisome number and function, and mitochondrial dysfunction [4]. Malnutrition can also lead to immune system impairment and increased mortality from infections [5–7]. Acute or chronic diseases and their treatment methods can also cause malnutrition, mainly due to altered metabolism [8]. The high prevalence of diabetes-related comorbidities and complications can further undermine nutritional status, and malnutrition can result in muscle function loss, delayed wound healing, reduced bone mass, immune system dysfunction, and decreased systemic function [9, 10]. A number of scores have been developed to assess human nutritional status, including the prognostic nutritional index (PNI), the controlled nutritional status (CONUT) score, and the nutritional risk index (NRI) [11–13].

In this article, we postulate that there is a strong correlation between the PNI and the development of PAD. To address this hypothesis, we prospectively investigated the relationship between the PNI and PAD using a nationally representative sample of adult PAD participants in the United States.

## Matieral and methods

### Study population

**Fig. 1:**
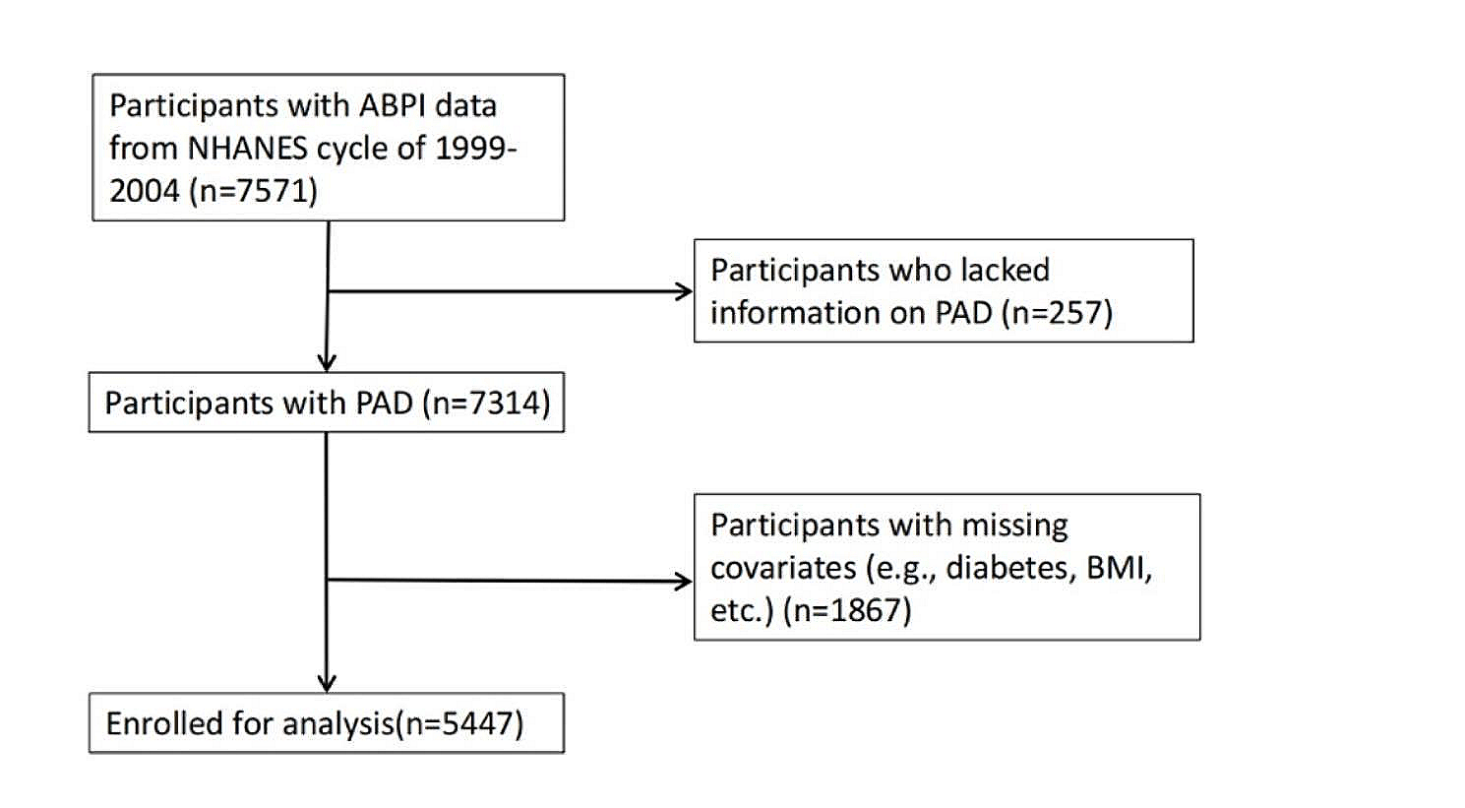
Flow chart of participant selection

The National Health and Nutrition Examination Survey (NHANES) is an ongoing research project that provides estimates of the population’s nutrition and health status in the United States. This survey uses a stratified, multistage probability design to recruit a representative sample of the American population [14]. Data is gathered through structured interviews with individuals at home, health screenings at mobile health screening centers, and laboratory sample analysis. In keeping with previous studies that have investigated PAD using the NHANES database, we analyzed data from the 1999–2004 NHANES cycles (*n* = 31,126). A total of 7571 participants aged 40 years or older had valid ankle-brachial index (ABPI) measurements. We excluded participants who lacked information on PAD (*n* = 257), and those who lacked data on relevant covariates, such as BMI, lymphocyte and albumin (*n* = 1867) [15–17]. Finally, 5447 participants were included in our study (Fig. 1).

### Exposure variable and outcomes

PNI was calculated by albumin count [g/l] + lymphocyte count (10^9^)×5. The methods used to derive complete blood count (CBC) parameters are based on the Beckman Coulter method of counting and sizing, (Chap. 7 of the NHANES Laboratory), and albumin was determined by bromcresol purple, the above two indicators were measured on the same day, and the values from the first day of testing were used. ABPI was determined in subjects over the age of 40 by trained health technicians at a mobile examination center. Participants lie supine on the exam table during the exam. Systolic pressure is measured on the right arm (brachial artery) and both ankles (posterior tibial arteries). Systolic blood pressure is measured twice at each site for participants aged 40–59 years and once at each site for participants aged 60 years and older. The presence of PAD was defined as left- or right-sided ABPI ≤ 0.9 [18, 19] .

### Covariates collection

Baseline characteristics of the participants were obtained through questionnaires and examinations, including sociodemographic (sex, age, ethnicity, and education) and lifestyle information (smoking status). Plasma total cholesterol, and CRP (C-reactive protein) were measured by standard biochemical methods. BMI is calculated by dividing weight (kg) by height (m) squared. Races are classified as non-Hispanic whites, non-Hispanic blacks, Mexican Americans, other Hispanics, or others. The education level is divided into below high school, high school, equivalent education, and college. Hypertension is defined as an ASBP or ADBP of 140/90 mmHg, as currently taking blood pressure medication, or as previously diagnosed by a doctor or other health professional. Diabetes was defined as fasting blood sugar > 7 mmol/L, random blood sugar ≥ 11.1mmol/L or A1c ≥ 6.5%, or use of hypoglycemic drugs, or diagnosed with diabetes. Poverty income ratio (PIR) is an index for the ratio of family income to poverty. Smoking is defined as smoking at least 100 cigarettes in life.

### Statistical analysis

Due to the complex sampling design of the NHANES database, analysis of the data required consideration of sample weights, clustering, and stratified analysis. Normally distributed data were expressed as standard deviations, while non-normally distributed data were expressed as medians and interquartile ranges. Categorical variables were expressed as percentages and analyzed using chi-square tests, while quartiles of PNI levels were identified based on the distribution of the study population. One-way ANOVA tests (for continuous variables with normal distribution), Kruskal-Wallis tests (for continuous variables with non-normal distribution), and chi-square tests (for categorical variables) were used to compare differences between the four groups. logistic regression was used to estimate odds ratios (ORs) and 95% confidence intervals (CIs) for cardiovascular disease mortality associated with PNI.

We also conducted several sensitivity analyses to assess the robustness of our findings. In stratified analyses, we divided participants based on age (≤ 60 or > 60 years), gender (male or female), smoke (yes or no), diabetes (yes or no), BMI (> 28 or ≤ 28 kg/m^2^), and hypertension (yes or no). We assessed the significance of the interaction by examining the *P*-value of the product term between the PNI level and the stratified variables. All analysis was performed by R software (version 4.1, Vienna, Austria) and spss25.0 (Chicago, IL, USA), and *P* < 0.05 on both sides was significant.

## Results

### Characteristics of the study population

The baseline characteristics of the included participants are presented in Table 1. Significant differences were observed across each quartile of the PNI. Compared to participants with higher PNI scores (quartiles 3 and 4), those with lower PNI (quartiles 1 and 2) were more likely to be older, female, and non-smokers. These participants also had higher CRP levels and higher PIR levels. Furthermore, we observed that participants with lower PNI quartiles tended to have higher rates of PAD (Q1: 9.9%, Q2: 4.8%, Q3: 6.3%, and Q4: 5.6%, *P* < 0.001). Similar results were observed for the prevalence of diabetes and hypertension.

**Table 1:** Characteristics of the Study Population by PNI Quartile (Q)

Variable	Total(*n* = 5447)	Q1(*n* = 1451)	Q2(*n* = 1516)	Q3(*n* = 1252)	Q4(*n* = 1228)	*p*-Value
Age(years)	59.56 ± 13.10	63.12 ± 13.41	60.28 ± 13.48	57.76 ± 12.49	56.31 ± 11.64	< 0.001
Male,n (%)	2820(52.8)	686(47.4)	821 (53.8)	652(52.2)	661(53.9)	0.002
Hypertension,n (%)	2200(40.4)	667(46.1)	580(38.0)	469(37.6)	484(39.5)	< 0.001
Diabetes,n (%)	679(12.5)	218(15.1)	175(11.5)	122(9.8)	165(13.4)	0.008
**Education level n (%)**						0.003
Less than high school	1753(32.2)	443(30.6)	460(30.1)	405(32.4)	445(36.3)	
High school diploma or GED	1268(23.3)	348(24.1)	346(22.7)	281(22.5)	293(23.9)	
More than high school	2426(44.5)	655(45.3)	720(47.2)	563(45.1)	488(39.8)	
**Race, n (%)**						< 0.001
Mexican American	1169(21.5)	221(15.3)	313(20.5)	312(25.0)	316(26.0)	
Other Hispanic	213(3.9)	41(2.8)	60(3.9)	59(4.7)	53(4.4)	
Non-Hispanic white	3028(55.6)	864(59.8)	866(56.7)	678(54.3)	616(50.7)	
Non-Hispanic black	889(16.3)	291(20.1)	240(15.7)	175(14.0)	182(15.0)	
Other races	148(2.7)	29(2.0)	47(3.1)	25(2.0)	47(3.9)	
Smoking, n (%)	2917(53.6)	706(48.8)	816(53.5)	665(53.2)	730(59.5)	< 0.001
PIR	2.82 ± 1.61	2.85 ± 1.59	3.02 ± 1.73	2.80 ± 1.62	2.70 ± 1.64	0.001
BMI kg/m^2^	28.44 ± 5.85	28.48 ± 6.54	28.88 ± 6.07	27.90 ± 5.12	28.39 ± 5.33	0.001
CRP mg/L	0.37 ± 0.36	0.44 ± 0.39	0.37 ± 0.36	0.34 ± 0.35	0.35 ± 0.35	< 0.001
PAD	363(6.7)	143(9.9)	72(4.7)	79(6.3)	69(5.6)	< 0.001

Values are given as mean ± standard deviation or numbers and percentages. Q1: PNI ≤ 50.00; Q2: 50.01–53.00; Q3:53.01–56.00; Q4: PNI > 56.00. BMI, body mass index; CRP, C-reactive protein; GED, general educational development; PIR, poverty income ratio; PAD, peripheral arterial disease

### Association between PNI and PAD

Table 2 presents the results of the one-way logistic regression analysis for PAD, and it can be observed that there are statistically significant differences except for BMI and Gender. Table 3 displays the results of the multivariate logistic regression analysis. When PNI was used as a quartile-based categorical variable and the fourth quartile was used as a reference, participants in the first quartile had a higher risk of PAD in all three models. After adjusting for potential confounding variables, the OR and 95% CI of the first quartile of PAD were 1.593(1.232–1.991).

**Table 2:** One-way logistic regression analysis of PAD

Variables	Beta	S.E	Z	OR (95%CI)	*P*
Age	0.07	0.01	16.02	1.07 (1.06–1.08)	< 0.001
BMI	-0.02	0.01	-1.87	0.98 (0.96–1.00)	0.058
PIR	-0.27	0.04	-7.44	0.76 (0.71–0.82)	< 0.001
CRP	0.17	0.05	3.60	1.19 (1.08–1.31)	< 0.001
HDL	-0.01	0.00	-1.78	0.99 (0.99–1.00)	0.075
RBC	-0.50	0.11	-4.39	0.61 (0.49–0.76)	< 0.001
HGB	-0.16	0.04	-4.30	0.86 (0.80–0.92)	< 0.001
Gender					
Male				1.00 (Reference)	
Female	-0.05	0.11	-0.55	0.95 (0.77–1.18)	0.580
Race					
Mexican American				1.00 (Reference)	
Other Hispanic	0.10	0.33	0.22	1.10 (0.58–2.09)	0.762
Non-Hispanic white	0.30	0.15	1.99	1.35 (1.01–1.82)	0.047
Non-Hispanic black	0.62	0.18	3.38	1.85 (1.31–2.62)	< 0.001
Other races	-0.67	0.52	-1.32	0.51 (0.18–1.43)	0.203
Education level					
Less than high school				1.00 (Reference)	
High school diploma or GED	-0.23	0.14	-1.61	0.81 (0.61–1.04)	0.089
More than high school	-0.73	0.13	-5.63	0.48 (0.37–0.61)	< 0.001
Hypertension					
Yes				1.00 (Reference)	
No	-0.97	0.11	-8.53	0.38 (0.31–0.47)	< 0.001
Diabetes					
Yes				1.00 (Reference)	
No	-0.84	0.13	-6.39	0.44 (0.34–0.57)	< 0.001
Smoking					
Yes				1.00 (Reference)	
No	-0.73	0.12	-6.23	0.49 (0.39–0.61)	< 0.001
PNI					
Q1				1.00 (Reference)	
Q2	-0.80	0.15	-5.18	0.45 (0.34–0.61)	< 0.001
Q3	-0.49	0.15	-3.40	0.62 (0.46–0.82)	< 0.001
Q4	-0.61	0.15	-4.08	0.54 (0.40–0.73)	< 0.001

BMI, body mass index; CRP, C-reactive protein; GED, general educational development; PIR, poverty income ratio; PAD, peripheral arterial disease; HDL: high density lipoprotein; HGB: haemoglobin

**Table 3:** Odds ratio and 95%CI of the PNI for PAD

PNI	Model1		Modle2			Modle3	
	OR(95%CI)	*p*	OR(95%CI)	*p*	OR(95%CI)		*p*
Q4	Reference		Reference		Reference		
Q3	1.203(0.778–1.865)	0.173	1.167(0.762–1.375)	0.801	1.041(0.733–1.477)		0.673
Q2	1.514(0.098–1.342)	0.061	1.137(0.723–1.454)	0.367	1.153(0.835–1.590)		0.387
Q1	1.651(1.421–2.133)	0.005	1.609(1.267–2.042)	< 0.001	1.593(1.232–1.991)		< 0.001

Data are presented as odds ratios, 95% CIs (confidence intervals), and *p*-value. Model 1 adjusted for age, sex, and race. Model 2 adjusted for age, sex, race, education level, poverty income ratio, body mass index, smoke status, hypertension, diabetes. Model 3 adjusted for age, sex, race, education level, poverty income ratio, body mass index, hypertension, diabetes, c reactive protein, high density lipoprotein, red blood cell, hemoglobin. PNI, prognostic nutritional index; PAD, peripheral arterial disease.

We also used Spearman correlation analysis to examine the relationship between covariates linked to PNI. As shown in Table 4, PNI was negatively correlated with age and PIR (*r* = -0.084 and − 0.006), and positively correlated with CRP and TC (*r* = 0.145 and 0.139).

**Table 4:** Correlations of PNI with some covariates

	PNI	
	r	*p*-Value
Age	-0.084	< 0.001
PIR	-0.006	0.671
CRP	0.042	0.002
TC	0.145	0.002

PNI: prognostic nutritional Index; CRP, C-reactive protein; PIR, poverty income ratio; TC, total cholesterol

### Stratification analysis

**Fig. 2:**
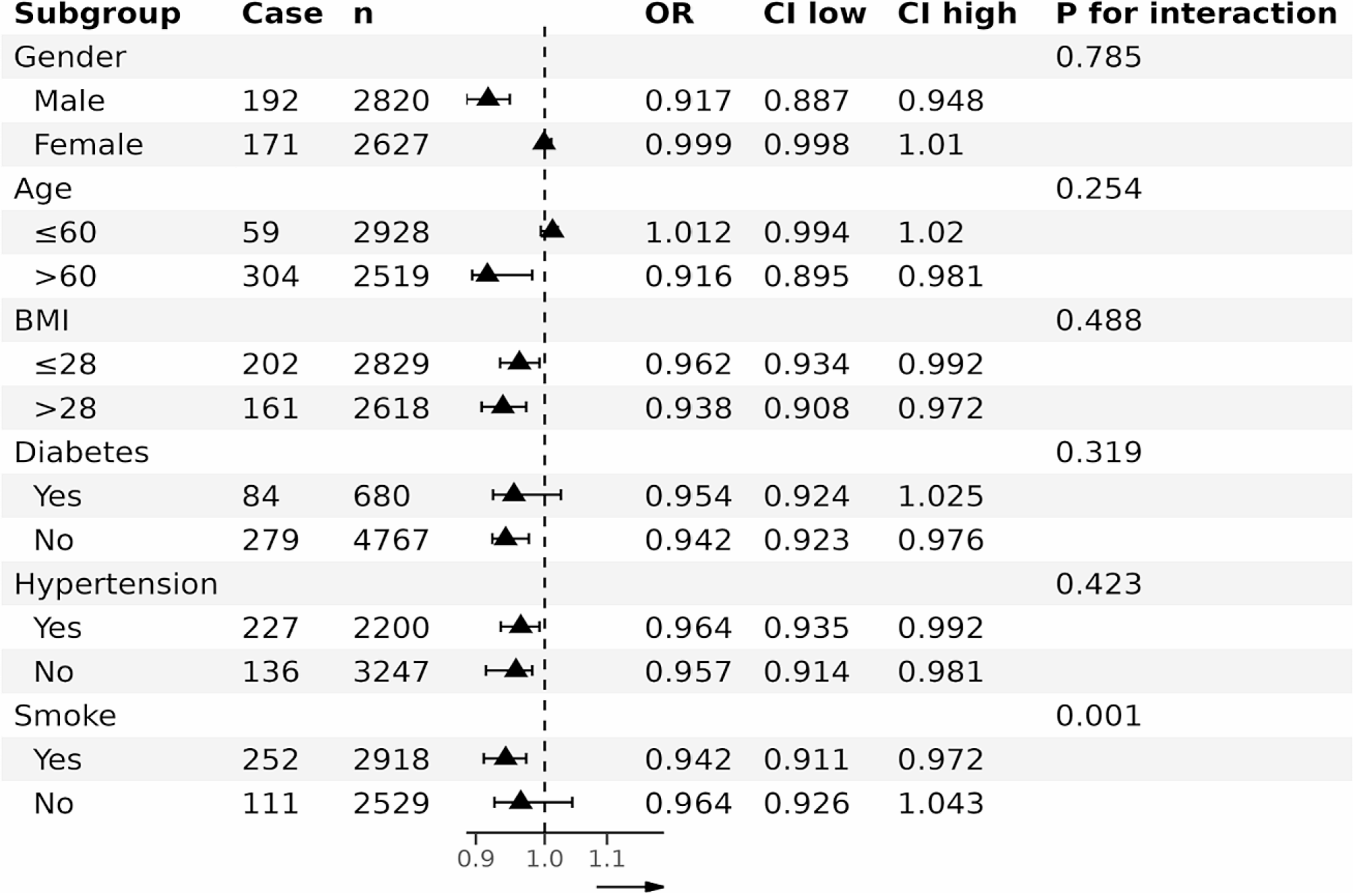
Subgroup analysis of association of PNI with PAD. CVD, cardiovascular disease; PNI:prognostic nutritional Index;PAD: peripheral arterial disease

Stratified subgroup analysis was performed by age, gender, hypertension, diabetes, and BMI. PNI was further treated as a continuous variable. Figure 2 shows that there was a negative correlation between PNI and PAD in participants older than 60 years, without diabetes, male, female, with a BMI greater than 28 or less than 28, smokers, hypertension, without hypertension, diabetes and without diabetes. Furthermore, the link between PNI and PAD was stronger in smokers (P for interaction = 0.001).

## Discussion

In this large cross-sectional study, a significant positive correlation was observed between PNI and PAD prevalence, suggesting that lower PNI may lead to a high risk of PAD. We observed a sharp increase in the risk of PAD when PNI was below 50. Atherosclerosis is a systemic inflammatory disease [20]. Inflammatory markers such as NLR (Neutrophil-lymphocyte ratio), PLR (Platelet-lymphocyte ratio) and CRP can be used to predict cardiovascular risk in patients with coronary artery disease. Rein [21] et al. reported that patients with PAD have a higher incidence of systemic inflammation than patients with coronary artery disease. PAD is not simply a peripheral atherosclerotic disease, but part of a multivascular disease [22]. Recently, the concept of a malnutrition-inflammation-atherosclerosis syndrome has been proposed, where inflammation tends to promote a catabolic state that inhibits protein synthesis and stimulates protein degradation, leading to malnutrition and reduced GNRI [23]. Furthermore, malnutrition is a complex state that includes a decrease in protein reserves and caloric depletion, which can weaken immune defences. Reduced immune defences are a key factor in the development of many chronic diseases. Thus, there may be a positive feedback loop between inflammation, malnutrition, immune defences and adverse events, resulting in a vicious cycle [24]. PNI is a nutritional indicator that was originally developed to predict morbidity and mortality after gastrointestinal surgery [25]. Because the original PNI was complex and difficult to use routinely in clinical practice, Onodera et al. simplified its calculation method to be based on serum albumin levels and peripheral blood lymphocyte counts [26]. It is now one of the easiest routine indicators to measure. Several studies have found a significant association between PNI and atherosclerotic disease [27].

Although the significance of PNI for PAD has been reported, studies have been hampered by small sample sizes and low statistical power. Erken conducted a retrospective study that included 266 patients with peripheral arterial disease divided into an amputation group (*n* = 39) and a non-amputation group (*n* = 227). The results found that PNI was lower in the amputation group compared to the non-amputation group (31.8 vs. 39.4), and that immunonutritional status based on PNI was independently associated with amputation in patients with lower extremity PAD [13]. Itagaki included 278 patients with PAD treated with endovascular therapy with major adverse cardiovascular events (MACE) as an outcome at 5 years, and found that PNI was associated with a risk of MACE and possessed good prognostic predictive power [28]. Compared with the previous studies mentioned above, our study examined PNI status in a larger population cohort and revealed the relationship between high or lower PNI and the development of PAD in the population. The importance of large-scale studies cannot be overemphasized, as small studies with null results are much more likely to remain unpublished than small studies with important results, leading to publication bias.Previous retrospective studies have demonstrated that PNI can predict post-surgical clinical outcomes in patients with atherosclerotic diseases other than PAD. In a study by Balun [29], 809 patients undergoing coronary stenting for coronary artery disease were included to investigate the correlation between PNI and post-procedural all-cause mortality and major adverse cardiovascular events. The results concluded that a low PNI value indicates poor nutritional status, which was thought to accelerate the inflammatory process leading to atherosclerosis and restenosis (ISR). This is in line with our own findings. Additionally, Wada et al. reported the correlation between PNI values and long-term clinical outcomes as well as percutaneous coronary intervention in patients with stable angina [30]. A total of 1988 patients were included in the study, which found that stable angina patients with lower PNI had an increased risk of developing major adverse cardiovascular events (MACE) compared to patients with higher PNI values. Furthermore, PNI was also significantly associated with long-term cardiovascular outcomes in patients with stable angina. These findings suggest that PNI may be a useful predictor of cardiovascular risk in patients with stable angina.

In this study, we observed that individuals with lower PNI were more likely to be elderly or patients with a larger BMI. It is not surprising that albumin may decrease in the elderly due to insufficient nutrient intake and increased loss of nutrients. As for BMI, previous studies have confirmed that obesity may play a role in the development and progression of PAD, so it is not surprising that obesity is more common in individuals with lower PNI [31]. In addition, Spearman correlation analysis showed that participants with lower PNI levels tended to have higher age, PIR, CRP values, which may also increase PAD burden in the general population. Subgroup analyses showed that the inverse association between PNI and PAD was similar in the population. However, we found that some subgroups, including older participants and smokers, had significantly lower ORs than the corresponding subgroups. This is due to aging and smoking moderating the effect of PNI on PAD, which needs to be validated in a larger specific population in the future. Previous studies with large amounts of data point out that the prevalence of peripheral artery disease increases with age [32, 33]. As for smoking, it is a well-established risk factor for atherosclerosis worldwide [34]. However, the reduced sample size after stratification could lead to potential bias, so we need to validate this result with a larger sample size.

Despite the important findings of our study, it is important to acknowledge certain limitations. Firstly, the cross-sectional study design means that causality cannot be determined. Therefore, longitudinal studies with large sample sizes are necessary to validate our results. Secondly, the lack of available ABPI data for subjects under the age of 40 limited our ability to analyze this association across various age groups. Thirdly, it is important to note that the results cannot be generalized to other populations with different demographics, as all participants were residents of the US. Lastly, the definition of PAD was based solely on ABPI measurements and was not confirmed by symptoms such as intermittent claudication, or findings from ultrasonography and angiography.

## Conclusions

In conclusion, our results suggest that lower PNI is associated with a higher risk of PAD in US adults. Our findings could provide valid information for large-scale prospective studies to further draw attention to nutritional status.

## Data Availability

The corresponding author (Yongquan Gu) will provide original data supporting the conclusions of this paper without reservation.
